# Evaluation of Fluorescence Detection Algorithms for Efficient ROI Setting in Low-Cost Real-Time PCR Systems

**DOI:** 10.3390/bios15090598

**Published:** 2025-09-10

**Authors:** Seul-Bit-Na Koo, Ji-Soo Hwang, Chan-Young Park, Deuk-Ju Lee

**Affiliations:** 1 Division of Software, Hallym University, Chuncheon-si 24252, Republic of Korea; rntmfqlcsk@hallym.ac.kr (S.-B.-N.K.); seattle@hallym.ac.kr (J.-S.H.); 2 Bio-IT Research Center, Hallym University, Chuncheon-si 24252, Republic of Korea

**Keywords:** fluorescence detection, Fresnel lens, image processing, open-platform camera, real-time PCR

## Abstract

This study proposes a region of interest (ROI) setting method to improve the accuracy and efficiency of fluorescence detection in a compact real-time multiplex fluorescence PCR system. Conventional commercial real-time PCR systems are limited in point-of-care (POC) environments due to their high cost and complex optical structures. To address this issue, we developed a low-cost, compact system using an open-platform camera and a Fresnel lens. However, in such a simply structured system, variations between the wells of the polymerase chain reaction (PCR) plate may affect the accuracy of fluorescence detection. In this study, after capturing images with a CMOS camera, we propose two ROI image processing algorithms. The proposed algorithms reliably extract fluorescence signals and compare ROI deviations caused by variations between wells to determine whether physical correction is necessary. To validate the system, we performed comparative analysis of real-time DNA amplification images and fluorescence dye images collected over multiple periods. Based on evaluations using manual detection as a reference, it was confirmed that even a simple algorithm can achieve stable fluorescence detection while minimizing ROI distortion. This study presents an efficient method for enhancing the accuracy of quantitative fluorescence analysis in small PCR systems and is expected to contribute to improving the performance of point-of-care diagnostics, thereby increasing accessibility to on-site diagnostics in the future.

## 1. Introduction

Despite advances in medical technology, infectious diseases continue to reemerge and mutate due to various factors, remaining a critical global issue [1]. A significant portion of emerging infectious diseases are zoonotic, transmitted from animals to humans, and often develop into widespread outbreaks, severely impacting global health and economies [2,3]. These characteristics make the diagnosis of infectious diseases challenging, particularly in resource-limited settings. Real-time polymerase chain reaction (real-time PCR) has become an essential tool in the diagnosis of infectious diseases due to its high sensitivity and specificity [4,5,6]. This technology relies on accurately detecting fluorescent signals based on nucleic acid amplification. The fluorescent signals collected during amplification cycles serve as crucial data for fluorescence analysis in quantitative assessments. The accurate detection of fluorescent signals is essential for generating reliable analytical results [7,8,9]. Commercial real-time PCR systems reliably detect fluorescence signals using high-performance image sensors and complex optical architectures. However, their high cost and fixed-well configurations limit miniaturization and flexible deployment [10,11]. In particular, image-based region of interest (ROI) detection algorithms are typically not integrated, making it challenging to perform precise fluorescence analysis in low-cost systems [12]. Consequently, there is a growing demand for low-cost, simplified real-time PCR systems that can be utilized in PoC settings [13,14]. Accurate fluorescence detection methods are critical to ensure the performance of these systems. To accurately detect fluorescent signals, it is essential to detect consistent Regions of Interest (ROI) in each amplification cycle [15,16]. However, physical and environmental variations can cause alignment errors between wells within the device, reducing the accuracy of fluorescence signal detection [17]. Commercial devices address this issue through periodic calibration, ensuring data consistency and quantitative performance [18,19]. In the field of biomedical imaging, various strategies have been developed for automatic region of interest (ROI) detection. Traditional methods such as edge detection, clustering, and template matching have provided the foundation for identifying target regions. More recently, deep learning approaches including lightweight convolutional neural networks (CNNs), YOLOv5, YOLOv7, U-Net, Vision Transformers (ViTs), and Grad CAM have shown strong performance in ROI detection tasks [20,21,22]. However, these methods often require substantial computational resources and specialized hardware, making them less suitable for resource-constrained point-of-care (PoC) devices [23,24].

Previous studies have proposed image-based fluorescence visualization methods using edge detection and Hough transform in silicon chip-based qPCR systems, and high-dimensional image processing algorithms in digital PCR platforms [15,25]. While these approaches demonstrate the precision of image-based analysis, their reliance on custom-fabricated chip architectures and high-performance computational environments limits their applicability in low-cost embedded environments.

In contrast to data-driven learning-based techniques, this study introduces a lightweight and practical solution based on rule-driven geometric projection and filtering. This approach is specifically designed to suit embedded and low-cost applications. The distinction highlights the relevance of our method in scenarios where processing speed, system simplicity, and hardware limitations are critical design factors. In a previous study, we proposed a cost-effective real-time PCR system utilizing a low-cost thermal cycler, an open-platform camera, and a Fresnel lens [26]. In this study, we propose an ROI setting method to improve the reliability of fluorescence signal detection in this system and validate its effectiveness. The proposed method includes a stable and efficient ROI setting approach that accounts for well-to-well variations within the device, reducing deviations between wells and preventing fluorescence data loss while maintaining sensitivity. This study compares ROI detection methods using both manual and automatic algorithms and analyzes the performance differences between simple and complex algorithms in automatic detection. Approaches for fixing ROI or periodically detecting it based on the device’s variability are explored. Fluorescent data collected over a month were analyzed by comparing real-time amplified fluorescent images with fluorescent dye reagent images. Evaluation of the central position and area of the ROI confirmed that stable fluorescence detection could be achieved even with a simple algorithm. This study demonstrates that stable and consistent fluorescence detection is feasible in a low-cost real-time PCR system and presents an efficient ROI setting method that maintains reliability.

## 2. Materials and Methods

### 2.1. Equipment Description

**Figure 1:**
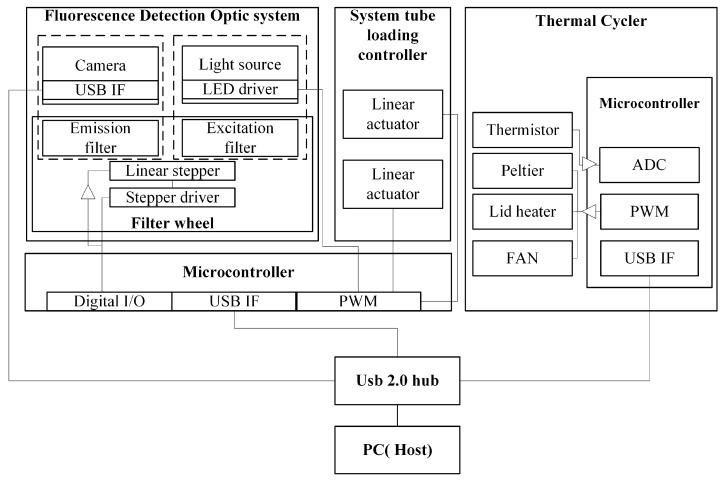
Block diagram of the real-time PCR device.

Figure 1 shows the block diagram of the real-time PCR equipment used in this study. The system is divided into two main parts: the amplification unit and the detection unit, both controlled by a microcontroller. These two components are arranged vertically. The amplification unit, which is a thermal cycling system, is the core part that performs the PCR and consists of a lid heater and a heating chamber. The lid heater heats the tube to a high temperature to prevent sample evaporation and allows fluorescence detection through holes aligned with the tube caps. Unlike conventional PCR equipment, the lid heater uses a thin aluminum plate and a flexible PCB substrate, enhancing miniaturization and efficiency while reducing production costs. The heating chamber consists of a heating block, Peltier element, cooling plate, heat sink, fan, thermally conductive lubricant, and a thermistor. The 25-well (5 × 5) aluminum heating block controls the temperature of the tubes containing the samples, while the heat sink, which uses a heatsink designed for computer components, reduces size and cost.

The detection unit, located above the thermal cycler, is an optical system consisting of a Fresnel lens, detector, light source, filter wheel, and motor. The fluorescence detection unit at the top is generally divided into an excitation part and an emission part. The excitation part includes an LED light source and an excitation filter, while the emission part includes a camera and an emission filter. In the proposed system, both the excitation and emission filters are integrated into the filter wheel, which also includes a motor and LED. The detector is an open-platform camera module that performs fluorescence detection.

A controller is used to load the heating block where the tubes are mounted, and an actuator is employed to mount the tubes. The actuator presses the lid heater and the detection unit onto the tubes to ensure proper alignment. The actuators perform position control functions based on signals from the microcontroller.

The device developed in this study was constructed with dimensions of approximately 16 cm × 27 cm × 23 cm and a total weight of 4.8 kg. It was designed with a simplified optical configuration and a full 5 × 5 tube array layout, offering a more compact and streamlined structure compared to conventional benchtop RT-PCR instruments.

**Figure 2:**
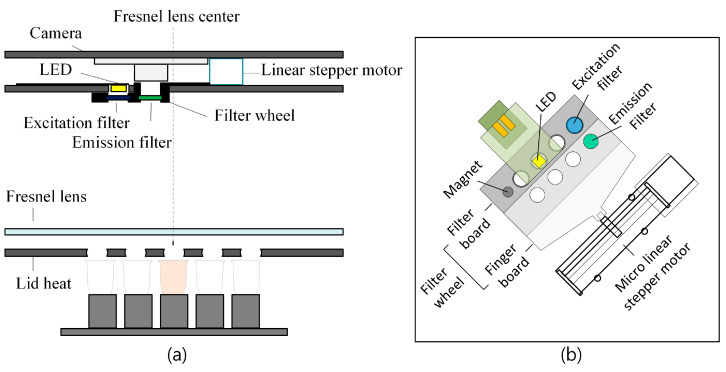
(**a**) Side view of the low-cost RT-PCR system; (**b**) system diagram of the filter wheel.

Figure 2a,b provide a detailed view of the structure of the filter wheel. To reduce system costs, the camera and light source are placed on the same plane, and both the center of the camera and the center of the Fresnel lens are jointly offset by 4.5 mm to minimize reflected light. The filter wheel contains emission and excitation filters for FAM, HEX, ROX, and CY5 fluorescence, and its position is controlled by a linear stepper motor. As the filter wheel rotates, it selectively positions the excitation or emission filter, either exciting the fluorescence or transmitting the emitted fluorescence to the detector. The camera and LED are fixed, and fluorescence is analyzed based on the movement of the filter wheel. A single white LED is used as the light source to uniformly illuminate the 25 wells, and the Fresnel lens is positioned at the focal distance between the lid heater and the optical system to prevent image distortion. The camera module is equipped with a Sony IMX298 image sensor, and the captured images can be processed by the host via USB communication. The host requires the circular ROI to be set based on the stored center points in order to measure the fluorescence intensity of each tube. Due to assembly tolerances, the center points of the tubes may vary between devices, which can cause variation between wells.

### 2.2. ROI Detection Considerations

**Figure 3:**
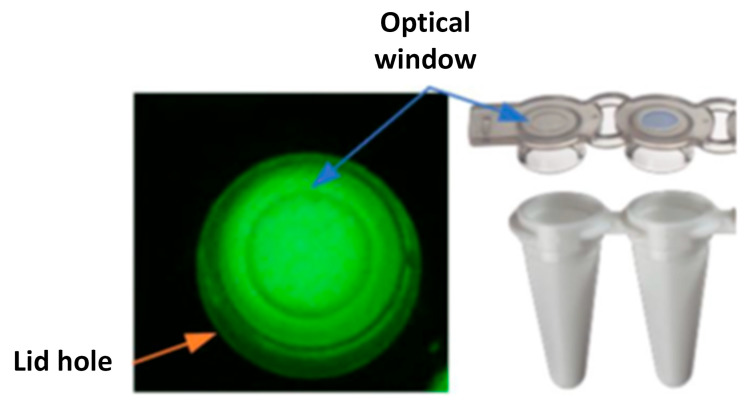
Position of the optical window and lid hole.

To ensure accurate quantitative analysis, it is crucial to optimize the detection of the region of interest (ROI). In our proposed system, amplification is conducted by inserting tubes into a heating block arranged at regular intervals, and fluorescence signals are detected through an optical window located on the top surface of the tube. At this time, it is essential to align the holes in the lid heater with the optical windows of the tubes to set the ROI. As shown in Figure 3, the tube cap has an optical window with a 3 mm diameter, and by aligning this with the lid holes, fluorescence can be precisely monitored.

The two main considerations when detecting the ROI are the variability within the device and the corresponding calibration of the system. Variability in the system can arise from physical, mechanical, and environmental factors, which are critical to accurate ROI detection and can cause misalignment between the essential components, such as the tubes and the lid heater. For instance, physical factors like thermal expansion or contraction of the tubes may lead to misalignment between the tubes and the heating block during the experiment. Mechanical errors may occur during tube insertion or the initial setup of the device. Additionally, environmental factors such as vibrations or temperature fluctuations during long-term experiments can cause internal module shifts, affecting alignment. To address such variability, markers can be placed on the lid heater plate to identify tube positions at each cycle, ensuring accurate ROI determination.

The second consideration is how to calibrate the device when there is no variability. If the changes in tube position are small enough to neglect device variability, the ROI can be detected using a calibration plate during the initial calibration. In subsequent experiments, the ROI can be fixed without the need for additional alignment steps, ensuring consistent measurements throughout the use of the device. While manually setting the ROI offers high accuracy, it can be time-consuming. On the other hand, automatic ROI detection algorithms can provide faster and more efficient solutions depending on the situation. Both complex and simple algorithms can be considered for automatic ROI detection, and in certain cases, simpler algorithms may offer faster and more reliable results.

**Figure 4:**
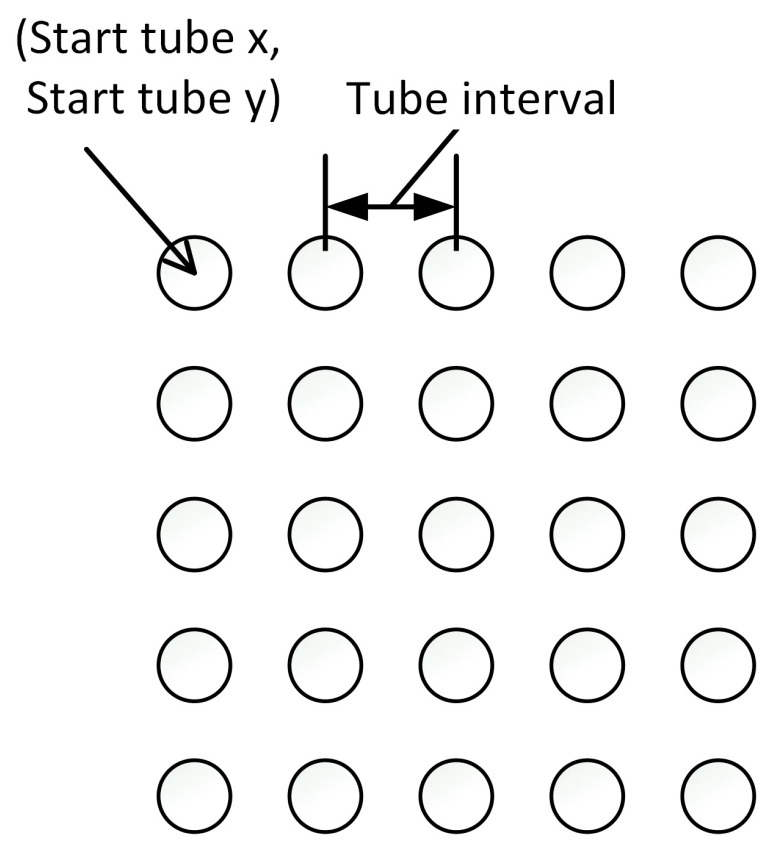
Consistent tube spacing.

As shown in Figure 4, the spacing between the tubes is uniform in the proposed system. Therefore, if the spacing of the 5 × 5 tube array is consistent, the ROI can be easily determined using the position and spacing of the (1,1) tube. Through this process, we propose a method for accurately detecting the ROI of the tubes and calibrating the device while considering variability.

### 2.3. Image Processing for Fluorescence Detection

The images acquired using the IMX298 camera (Arducam, Nanjing, China) were processed through a Python-based (version 3.11.9) pipeline on a desktop equipped with an Intel Core i7-10700 CPU (Intel, Santa Clara, CA, USA) and 32 GB of RAM (Samsung Electronics, Pyeongtaek, Republic of Korea). Actuator (OEM model, manufactured in China) control and sensor operations were managed by a microcontroller built on the Adafruit M0 (Adafruit industries, New York, NY, USA) platform.

**Figure 5:**
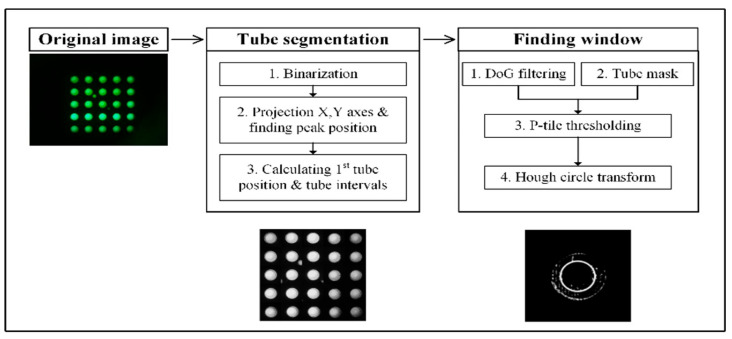
Image processing steps for correcting the ROI position of each tube in the fluorescence image using FAM.

Figure 5 shows the image processing steps for aligning the ROI position of each tube in the fluorescence image using the reference fluorescent dye FAM. The image processing steps for finding the center of the tube are divided into the “Tube grid find” and “Tube window find” stages. In the first step, the Tube Grid Find stage, a simple algorithm is used to estimate the center points of the 25 tubes and determine the boundary regions of each tube in the fluorescence image. In this process, the binarized fluorescence image is projected onto the X-axis and Y-axis, and the peaks of the generated signals are detected. A median filter is used to detect stable peaks, and a height parameter is set to determine the center position of the tubes. This method detects five horizontal and vertical peak points from the signal and calculates the approximate position of the first tube based on the average horizontal and vertical spacing between the projected peaks and the tubes.

To ensure stable peak detection, the tubes in the 5th row and column, which exhibit weaker fluorescence, were excluded. Based on experiments with a 4 × 4 tube array to mitigate noise and artifacts, the filter size was set to 33 pixels and the maximum height parameter to 27 pixels. The center of the first tube was selected by moving 1.5 times the tube spacing in the left diagonal direction from the average position at the center of the 4 × 4 tube array. This adjustment helps minimize overlap between adjacent tubes and improve performance. The tube spacing was calculated by averaging the distances of the four sides of the square formed by connecting the centers of the four corners.

The second step, Tube Window Find, uses a complex algorithm to detect the optically transparent window of the qPCR tubes based on the center points obtained from the Tube Grid Find step. Initial tube position estimation is performed using a simple algorithm, followed by periodic processing of the local region. This process involves four main tasks. First, a Difference of Gaussian (DoG) filter is applied to remove image details and extract the overall shape of the tube. User-defined parameters, such as the sigma value of the filter, are adjusted to optimize detection accuracy. Second, the Otsu binarization algorithm is used to automatically identify key points in the histogram and provide an outline of the bright parts of the tube. Then, a mask of the inner region of the tube is generated by eroding the edges of the image. Third, the p-tile threshold algorithm is used to selectively extract edge information from the masked region based on the DoG filter results. This step focuses on extracting only the tube window information to improve detection accuracy. Finally, the Hough circular transform algorithm is applied to accurately identify the circular edges and the center point of the tube.

### 2.4. Validation and Comparison of Fluorescence Detection Methods

In this study, the performance of fluorescence detection methods was validated and compared using two algorithms. The first algorithm is a complex fluorescence detection algorithm with high precision, while the second is a simpler algorithm that offers faster processing and efficiency. To evaluate the performance of both algorithms, the variability of the center points derived from each algorithm was assessed against manually obtained reference points. Additionally, the size of the ROI was converted into pixels to establish an acceptable range of variability.

As the first approach, the performance of the complex fluorescence detection algorithm was validated by comparing it with the manual fluorescence detection method. The manual detection was performed by extracting information for each tube based on the four corners of the image, and this was compared with the optical window information detected by the complex algorithm. Additionally, to evaluate the performance of the complex algorithm, all images were visually inspected.

As the second approach, the efficiency of the simple fluorescence detection algorithm was evaluated by comparing it with the complex algorithm. The difference between the center of the optical window obtained from the complex algorithm and the center of the tube obtained from the simple algorithm was measured, and the fluorescent regions of the tube centers extracted by each algorithm were compared. During this process, it was verified whether fluorescence detection could be achieved using the simple algorithm based on the position and spacing of the first tube.

The data used for the comparative experiments consisted of five reference fluorescence FAM images and fifteen amplification experiment images (PCR plateau stage images) collected over the course of one month. These images were used to assess various factors of variability during the experiments, allowing for a performance comparison of each algorithm. The fluorescent reference image was obtained using the fluorescent reporter 6-carboxyfluorescein (FAM). As the standard material, Chlamydia trachomatis (CT) was prepared at a concentration of 5 copies. The PCR mixture (total volume: 20 μL) consisted of 10 μL Master Mix, 5 μL Primer Mix (500 nM), 3 μL Probe Mix (250 nM), and 2 μL sterile distilled water. The thermal cycling conditions were as follows: initial denaturation at 97 °C for 2 min, followed by 45 cycles of 97 °C for 10 s and 60 °C for 20 s. The real-time PCR amplification curve was generated by applying the fixed ROI value determined from the optimal algorithm, selected through the performance comparison of multiple algorithms. This experiment verified the stability of the selected algorithm under actual experimental conditions.

To evaluate and validate the performance of the fluorescence detection algorithms, we conducted a statistical comparison of the positional deviations among manual detection, the complex algorithm, and the simple algorithm. The dataset used for analysis comprised a total of 20 fluorescence images—five baseline FAM images and fifteen post-amplification images—each containing 25 wells, resulting in 500 data points (n = 500).

For each well, the differences in the center coordinates between algorithms were calculated in pixels and subsequently converted to millimeters using a spatial resolution of 52.3 pixels per millimeter.

The normality of the coordinate difference distributions was assessed using the Shapiro–Wilk test. Since some distributions did not satisfy the assumption of normality, the non-parametric Wilcoxon signed-rank test was applied to the paired x- and y-coordinate differences. All tests were two-sided, with a significance level set at α = 0.05.

Results were reported as the mean ± standard deviation, and all *p*-values were presented as exact values. Statistical analyses were performed using the SciPy library in Python.

## 3. Results

### 3.1. Image Processing Results

#### 3.1.1. Parameters for Tube Grid

The simple algorithm for finding the tube grid requires parameter settings to roughly locate the centers of the tubes. While the images used for tube detection contain a 5 × 5 array, the tubes in the 5th row and 5th column appear darker due to optical reasons. Therefore, to obtain stable results, the parameters of the algorithm were adjusted based on the corner positions of the tubes in the 4 × 4 array.

**Figure 6:**
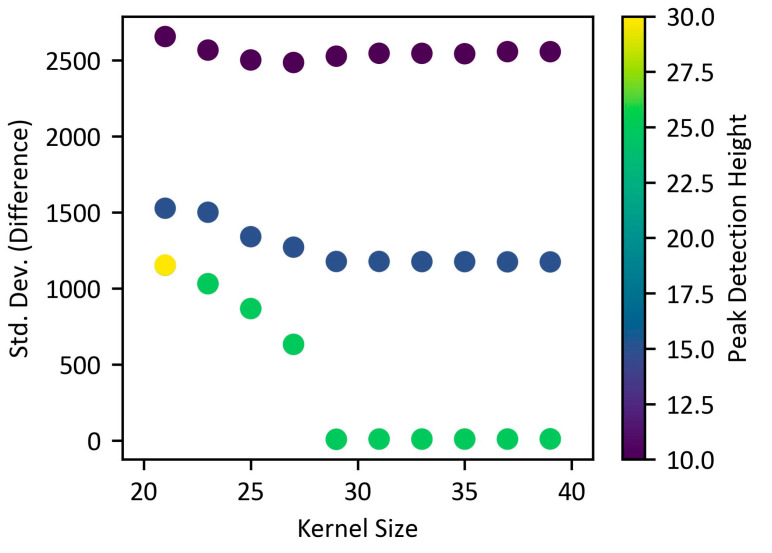
Distribution of standard deviation according to median filter size and peak height.

Figure 6 presents the results of the process for roughly determining the parameters required to find the tube grid in the images. Experiments were conducted on 20 images by varying the median filter size from 21 to 40 and the peak height from 10 to 60. For each combination, the standard deviation of the maximum deviation between the 4 × 4 corner peak positions and the actual positions was calculated.

As a result, a pattern of decreasing standard deviation values was observed when the median filter size was 29 or greater and the peak height was 20 or higher. This suggests that these parameter combinations are more effective at finding stable peaks in the images. Specifically, if a particular parameter combination failed to detect any peak values in an image, that combination was excluded from the overall standard deviation calculation. When the peak height was 30 or lower, peak values were successfully detected in all images.

**Figure 7:**
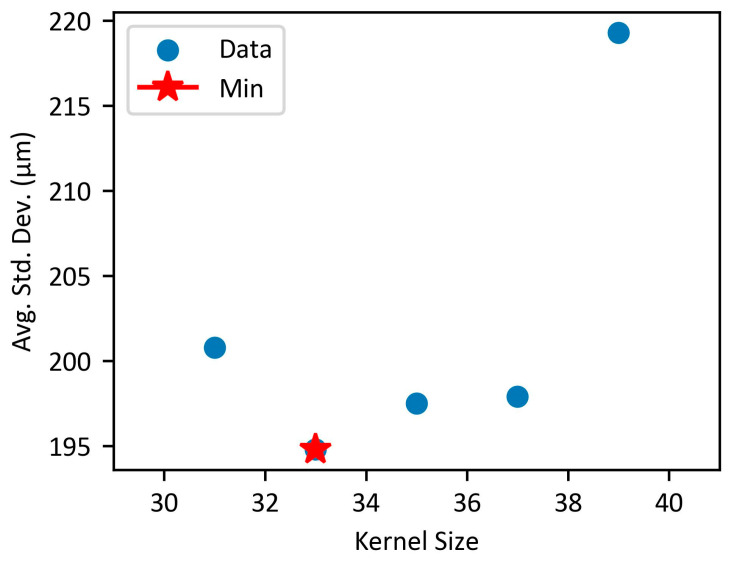
Change in average standard deviation according to median filter size in the optimized range.

Figure 7 shows the results of adjusting the median filter while increasing the peak height from 20 to 30 in increments of 1 to determine the positions of the tubes more accurately within the image. Based on previous results, the average standard deviation for each median filter was calculated only when the peak height was approximately 30 and the peak value was below 500. As a result, it was found that when the median filter size was 33, the average of the maximum standard deviations across all peak heights was the smallest, at 0.195 mm. When the peak height was 27, it showed the smallest standard deviation while also achieving the highest peak height value.

#### 3.1.2. Tube Grid Results Using the Simple Algorithm

**Figure 8:**
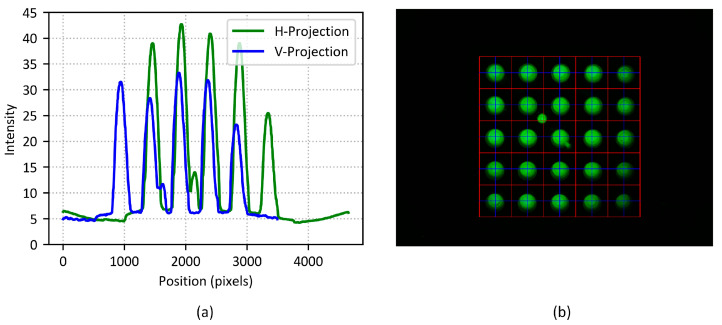
(**a**) Peak graph of the fluorescence image with the simple algorithm applied; (**b**) fluorescence image with 5 × 5 tube array grid. Blue lines indicate estimated tube centers from the simple algorithm; red lines mark tube boundaries.

Figure 8a shows the peak graph derived from the fluorescence image after applying the simple algorithm, following the determination of the two parameters. The graph represents the five peak points on both the X and Y axes, corresponding to the tube positions in the first row and first column within the image.

Figure 8b visually represents the grid that determines the positions of the entire 5 × 5 tube array, based on the stable spacing and the first position information. The position and spacing of the first tube were derived by averaging the 4 × 4 corner position information with stable brightness from five reference fluorescence images. The positions of all tubes are visually displayed with a grid overlay, where the blue lines represent the detected tube positions, and the red lines indicate the boundary regions of each tube. Although the blue lines of the grid do not perfectly align with the center of the optical windows for some tubes, they remain stably within the tube boundaries, indicating that the positions were accurately detected.

#### 3.1.3. Optical Window Detection Results Using the Complex Algorithm

**Figure 9:**
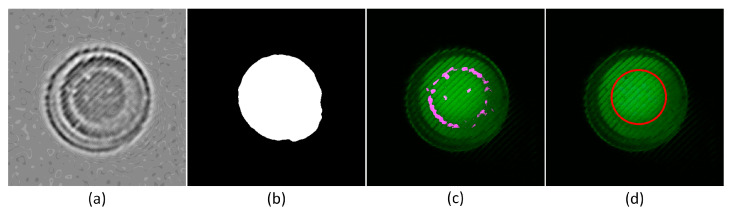
Sequential results of detecting the optical windows of the tubes based on information obtained through the simple algorithm: (**a**) dog; (**b**) window; (**c**) edge; (**d**) fitted circle.

Figure 9 shows the step-by-step results of detecting the optical windows of the tubes, based on the approximate tube center and spacing information obtained through the simple algorithm. Each step is carried out using the complex “Tube Find” algorithm, illustrating the process of progressively detecting the tube window locations more accurately. Figure 9a shows the result of applying a Difference of Gaussian (DoG) filter to extract the overall shape of the tubes. Two Gaussian filters were used to remove the concentric circle noise caused by the Fresnel lens, with sigma values set to 5 and 7, respectively. After blurring the image with the filters, the image filtered with sigma = 5 was subtracted from the image filtered with sigma = 7, resulting in the overall shape of the tubes. Figure 9b shows the detection of the tube structure by applying the Otsu binarization algorithm to the green channel of the original image. At this stage, a mask was created by eroding the outer boundary by 15 pixels. In Figure 9c, p-tile thresholding was used to delineate the tube window boundary more precisely by selecting only the top 1% of edges within the mask. Finally, Figure 9d shows the result of detecting the tube windows as circles using the Hough Circle transform. Although the complex algorithm took longer to process compared to the simple algorithm, it was able to detect the tube window locations with greater precision. It effectively removed the concentric circle noise caused by the Fresnel lens and accurately identified the centers of the tube windows, improving the reliability of fluorescence detection.

To quantitatively compare the processing efficiency of the two fluorescence detection algorithms, we measured the average image processing time under identical hardware and software conditions. The simple ROI-based algorithm, which utilizes fixed tube spacing and coordinate estimation, processed each image in approximately 78 ms. In contrast, the complex algorithm, which involves multi-stage operations such as DoG filtering, thresholding, and Hough circle detection, required about 213 ms per image. Based on 20 fluorescence images, these results suggest that the simple algorithm may offer a more efficient alternative in scenarios where rapid real-time processing is essential.

### 3.2. Comparison of Fluorescence Detection Algorithms

#### 3.2.1. Validation of Manual Detection

To establish validation criteria, the accuracy of manual detection was evaluated using the ImageJ program. Measurements using ImageJ (version 1.53e) calculated a ratio of 52.3 pixels per millimeter to convert the images to actual distance.

**Figure 10:**
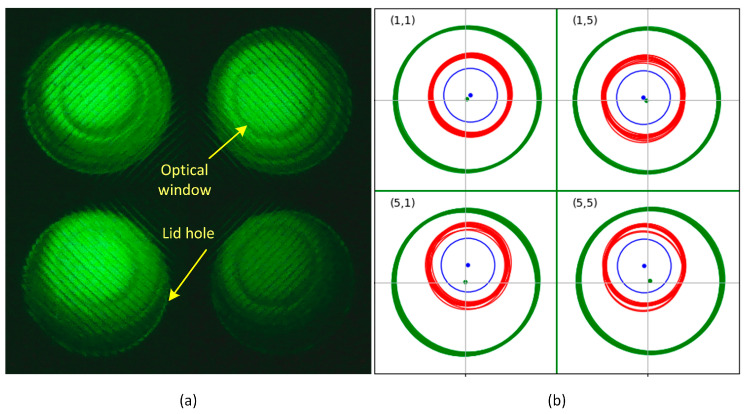
(**a**) Images of the four corner tubes. (**b**) Detected lid holes (green) and optical windows (red) in 15 corner images. Green and blue dots show average positions. Blue circle (50-pixel radius) is centered on the mean window position.

The accuracy of manual detection was assessed using the final 15 images of amplification performed on different days. Among them, the position information of the lid holes and optical windows for the corner tubes in the 5 × 5 array ((1,1), (1,5), (5,1), (5,5)), which exhibited significant distortion, was used. Figure 10a provides examples of images from (b) ROI detection result for each tube.

The four corner tubes, where brightness and contrast were adjusted by 10% to enhance visibility. To maintain image consistency, the images were cropped to a consistent size based on the averaged center of the lid hole for each corner, and then compared.

Figure 10b visually represents the positions of the lid holes and optical windows for the 15 images of the four corners. The lid holes are shown as green circles, and the optical windows as red circles. The average position of the lid hole is represented by a green dot, while the optical window’s average position is indicated by a blue circle and blue dot. From the average position, it can be observed that the lid holes are closely aligned with the grid center. For the optical windows, the blue dots for positions (5,1) and (5,5) deviate from the grid, but based on the average center point, when viewing the average circle drawn with a 50-pixel radius, they are sufficiently within the grid. Although the red circles representing the actual optical window area show some wobbling for (1,5), (5,1), and (5,5) compared to (1,1), they remain stably within the lid holes. The radius of 50 pixels was empirically chosen to consistently capture the central region of the optical window across all images, while minimizing the influence of background noise. This value proved to be robust even for distorted corner tubes, falling within acceptable positional tolerances, and offering a practical balance between spatial accuracy and image clarity.

Table 1 shows the results of evaluating the area and positional accuracy of the lid holes and optical windows through manual detection for the four corner tubes. In terms of area consistency, the mean relative standard deviation of the lid hole was 0.024, indicating very high structural consistency. The optical window had more variability, with a relative standard deviation of 0.039, but still maintained high precision. Regarding positional accuracy, the maximum center deviation of the optical window was twice that of the lid hole, but when converted to actual distance, it was a very small 296 μm.

Over the course of a one-month experimental period, no significant deviation was observed when manually comparing the reference fluorescence images with the endpoint amplification images under various conditions, including tube expansion, thermal fluctuation, mechanical insertion, and pressure. These findings suggest that the system maintains high performance and ROI stability without requiring frequent recalibration, and that ROI adjustment based on reference fluorescence images is feasible. Furthermore, this approach demonstrates that reliable ROI detection can be achieved through image-based algorithms alone, without relying on high-precision mechanical structures such as 96-well plates.

Based on this structural stability and experimental consistency, we statistically evaluated the positional accuracy of manual detection. A total of 15 amplification images containing the four corner tubes were analyzed. The deviations in the center coordinates of the optical windows remained within a maximum of 15.5 pixels (0.296 mm), and statistical analysis confirmed that the positional variation in manually detected centers did not exceed 0.3 mm.

Given that this deviation is negligible compared to the tube radius (1.5 mm), the manually selected centers provided a reliable and reproducible reference without the need for repeated calibration. These coordinates were subsequently used as ground truth data for evaluating the performance of the automatic detection algorithms.

#### 3.2.2. Evaluation of the Complex Algorithm

The performance of the automatic fluorescence detection method using the complex algorithm was evaluated by comparing it with the manual fluorescence detection method. The validation was conducted using the four corner images from the last 15 images of the amplification sequence.

The complex algorithm accurately calculated the center of the optical windows. Table 2 presents the tube position evaluation data between the two measurement methods. The unit is expressed in pixels. In terms of maximum displacement for each algorithm, the variability of the automatic algorithm was larger than that of the manual detection, but when converted to actual distance, the difference was 78 μm.

The maximum displacement difference between the two methods was 10.6 pixels (202 μm), which is not significantly different when compared to the maximum displacement in manual detection. The maximum position deviation between the centers of the circles in the manual detection was 15.5 pixels (296 μm), and the displacement difference with the complex algorithm falls within the acceptable margin of error. Additionally, considering that the deviation in radius is approximately 7.5 pixels (148 μm), the maximum displacement of 10.6 pixels (202 μm) between the center of the circle detected by the complex algorithm and the manual detection does not significantly affect the experimental results. This indicates that the automatic algorithm provides reliable performance compared to manual detection.

Finally, a comparison of the average displacement and variability ratio in manual detection showed that the complex algorithm demonstrated similar accuracy within an acceptable range. Therefore, the complex algorithm provided reliable results in an automated manner while maintaining performance similar to that of manual detection. A total of 20 images were used to visually verify the optical window areas through the complex algorithm.

**Figure 11:**
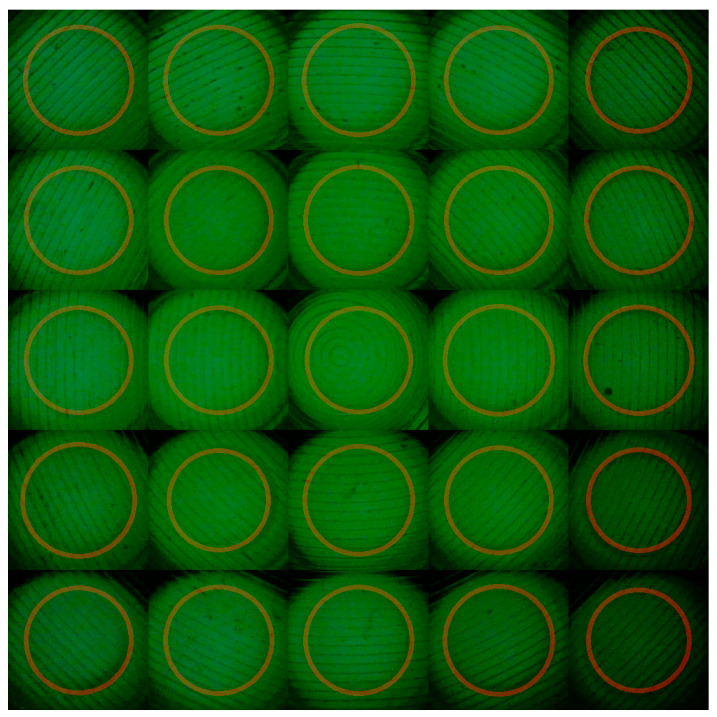
Optical window areas of 20 images using the complex algorithm.

Figure 11 shows the marking of the 25 optical windows using the complex algorithm with FAM fluorescence images. Visually, it can be confirmed that the optical windows were correctly marked.

Table 3 presents the statistical results of the complex algorithm. The maximum displacement of the tube positions across all 20 images was found to be 24.1868 pixels. The maximum displacement between the generalized tube positions, based on the average center coordinates and spacing of the 20 fluorescence images, was 24.93 pixels. The fact that both values are similar, approximately 24 pixels, suggests that the stability of the equipment was consistently maintained despite the experimental conditions. Additionally, this indicates that the analysis method using the complex algorithm provides consistent performance. In particular, comparing the averaged tube positions of the 5 fluorescence reference images with those of the 15 amplification images, the maximum displacement was found to be 16.29 pixels.

The relatively small positional deviation between the baseline fluorescence images and the amplification images supports the reliability of using baseline images, processed by the complex algorithm, for ROI determination.

To further assess the reliability of the complex algorithm, a paired statistical comparison was performed between manual detection and the complex algorithm, using 15 amplification images from the four corner wells (n = 60 data points). The mean positional difference between the two methods was 7.68 ± 3.88 pixels (approximately 0.147 ± 0.074 mm), and the Wilcoxon signed-rank test revealed no statistically significant difference between them (*p* > 0.05).

These findings indicate that the complex algorithm achieves comparable accuracy to manual detection within an acceptable margin of error, demonstrating that the automated approach can provide robust and reliable performance.

#### 3.2.3. Evaluation of the Efficiency of the Simple Algorithm

To evaluate the validation and efficiency of the automatic fluorescence detection method using the simple algorithm, it was compared with the automatic fluorescence detection method using the complex algorithm. After extracting the center and spacing of each tube through the simple algorithm using five fluorescence images, the generalized tube positions and spacing were determined based on the average center coordinates and spacing.

When calculating the displacement between the 25 optical window center coordinates obtained using the complex algorithm on a total of 20 images and the generalized center coordinates, the maximum displacement was found to be 28.5 pixels. The displacement calculated based on the averaged information extracted using the complex algorithm from the fluorescence reference images was 27.43 pixels. The maximum displacement difference between the two algorithms was only 20 μm, indicating that the displacement difference was not statistically significant. This result was consistently observed even in images with minor distortions or positional offsets, demonstrating that the simple algorithm can maintain reliable ROI detection under suboptimal imaging conditions. Therefore, considering both efficiency and performance, the use of the simple algorithm appears to be a better choice.

Additionally, the average radius of the optical windows obtained using the complex algorithm was 75 pixels, which, when converted to the actual optical window radius of 1.5 mm, becomes 78 pixels. This closely matches the actual tube radius and using the generalized grid coordinates obtained with the simple algorithm, the ROI was set with a radius of 50 pixels to confirm the differences between the tubes within the images.

This ROI size was adopted based on the manual detection validation described in Section 3.2.1, where it was found to consistently capture the central region of the optical window in peripheral tubes. Unlike the complex algorithm, which detects the full boundary of the optical window, the simple approach deliberately employs a smaller, fixed-radius ROI to reduce background noise and improve computational efficiency. This strategy also offers flexibility when dealing with varying tube sizes or in low-cost system environments. Despite the smaller ROI size, detection accuracy was not compromised.

**Figure 12:**
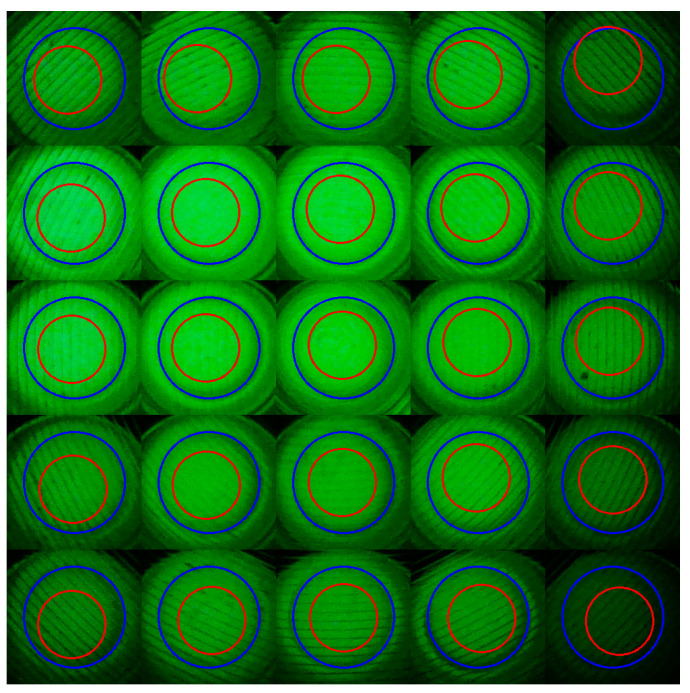
Optical window areas from the complex algorithm and ROI designated using the tube centers from the simple algorithm.

Figure 12 shows the optical window areas detected by the complex algorithm and the ROIs set with a radius of 50 pixels from the tube centers found using the simple algorithm. The blue circles represent the optical windows detected by the complex algorithm, while the red circles indicate the ROIs determined by the simple algorithm.

This indicates that the ROI region determined by the simple algorithm is well contained within the fluorescence detection area of the optical window, confirming that the algorithm provides sufficient reliability for accurate detection while maintaining low computational complexity.

Based on this visual and structural validation, we conducted a statistical evaluation of the positional accuracy of the simple algorithm in comparison with the complex algorithm. A direct comparison was performed using 20 fluorescence images, each containing 25 wells, resulting in 500 data points (n = 500). The mean positional difference between the two methods was 5.47 ± 3.12 pixels (approximately 0.105 ± 0.060 mm), and the maximum difference observed was 16.3 pixels (approximately 0.311 mm).

The Wilcoxon signed-rank test revealed a statistically significant difference in coordinate displacement between the two methods (dx: *p* ≈ 4.5 × 10^−38^; dy: *p* ≈ 9.4 × 10^−16^). However, the magnitude of this difference was very small compared to the tube radius (1.5 mm), indicating that it is practically negligible. These results support that the simple algorithm offers sufficient accuracy for ROI detection while providing superior computational efficiency.

Finally, the center and spacing statistics of the simple algorithm are presented in Table 4.

The deviation of the center of the 1st tube was found to be 0.06 mm, and the average spacing between the tubes was 9.05 mm, with a deviation of 0.05 mm between tubes. The actual distance between tubes in the heating block is 9.1 mm. These results suggest that the fluorescence brightness can be reliably measured using only the simple algorithm, based on the center and spacing of the 1st tube.

**Figure 13:**
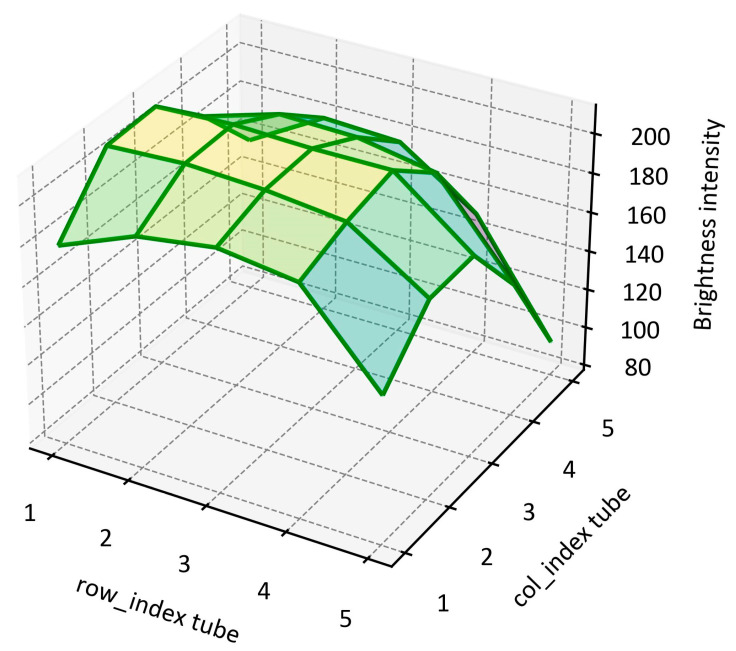
Average brightness of each well obtained from five images of the FAM fluorescence tray.

Figure 13 shows the average brightness of each well calculated from five images of a FAM fluorescence tray at a concentration of 750 femtomole/μL, which corresponds to the brightness measured at the point where fluorescence amplification begins. It was observed that the wells located in the 5th row and column, farther from the light source, tended to have reduced brightness compared to the other wells. This phenomenon is interpreted as a result of the attenuation of the fluorescence signal as the distance from the LED increases.

Additionally, despite uniform detection of the ROI, these brightness differences could potentially cause issues with uniformity in future quantitative analyses. When the fluorescence signal slightly decreases, additional correction may be required for accurate quantitative analysis.

**Figure 14:**
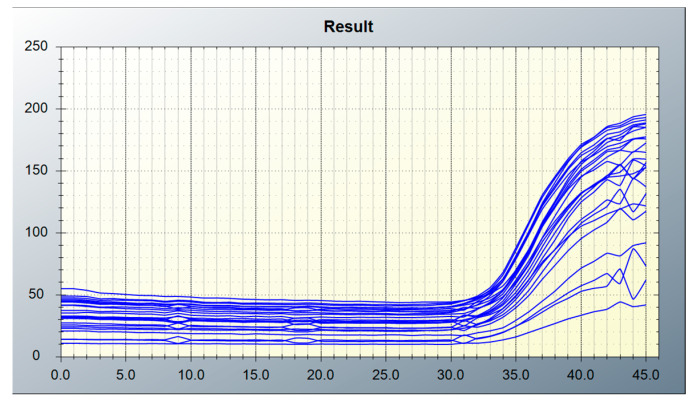
Fluorescence amplification curves from real-time raw data using fixed ROIs in 25 wells with the simple algorithm.

Figure 14 presents fluorescence amplification curves derived from real-time raw data collected from a 5 × 5 array of wells, using fixed ROIs determined based on the (1,1) position and tube spacing obtained through the simple algorithm. The horizontal axis represents the number of cycles during the amplification process, while the vertical axis indicates the fluorescence intensity measured at each cycle. The fluorescence intensity values were derived from image data acquired by the CMOS camera, processed in 8-bit format, and calculated based on pixel brightness values ranging from 0 to 255. The fluctuations seen in the curves reflect brightness variations caused by filter-related inconsistencies. Despite a reduction in signal observed in some peripheral wells, all amplification curves exhibit a comparable sigmoidal shape and threshold crossing points. The cycle thresholds (Ct values) are also distributed in similar positions across all wells, indicating that the simple ROI algorithm consistently detects the tube centers with sufficient reliability. These findings suggest that signal attenuation at the edges does not significantly compromise the measurement of Ct values or diagnostic accuracy. Nevertheless, we plan to explore additional correction or normalization strategies to improve the quantification of weak signals in future work.

## 4. Discussion

In this study, we evaluated and compared the performance of a simple ROI detection algorithm and a complex algorithm using a low-cost, compact RT-PCR device optimized for point-of-care (PoC) environments. Statistical validation revealed a significant difference in ROI coordinate displacement between the two algorithms (Wilcoxon signed-rank test, *p* < 0.001, n = 500), with a mean displacement of approximately 0.1 mm and a maximum displacement of approximately 0.3 mm. However, these values are minimal relative to the tube radius (1.5 mm) and are not expected to affect Ct estimation or diagnostic accuracy. These results demonstrate that the simple algorithm achieves sufficient reliability and stability while greatly reducing computational complexity, making it particularly well suited for embedded systems and real-time PoC diagnostic platforms.

Whereas conventional ROI detection methods often rely on expensive equipment, custom chip architectures, or high-performance computing resources, our rule-based geometric algorithm provides comparable accuracy with minimal hardware requirements. By integrating commercial PCR tubes, low-cost CMOS sensors, and lightweight computational techniques, this approach achieves both practicality and scalability in PoC applications.

During fluorescence acquisition, signal attenuation was observed in the edge wells of the 5 × 5 tube array. This was attributed to uneven illumination caused by the diagonal layout of the LED light source and the planar arrangement of the camera and LED board. Such signal degradation may impact quantification accuracy, particularly when scaling the system to larger formats such as 96-well plates.

To address this, future studies will explore a multilayered strategy combining optical optimization and image processing enhancements. One approach involves introducing depth offsets between the LED and camera to equalize the optical path. Another considers implementing a dual-frame image processing technique, where two images optimized for bright and dark regions are captured sequentially and merged to correct for intensity variation. On the analysis side, signal normalization algorithms will be developed to compensate for spatial intensity gradients, and modeling of overall fluorescence characteristics will be used to ensure consistent Ct value estimation.

These combined optical and algorithmic approaches are expected to complement each other, enabling uniform fluorescence detection and stable diagnostic performance without compromising structural simplicity or scalability of the system.

## 5. Conclusions

This study presented a low-cost, miniaturized RT-PCR system optimized for PoC environments and compared the performance of a simple ROI detection algorithm with that of a more complex approach. The simple algorithm achieved sufficient reliability and spatial accuracy while significantly reducing computational complexity, demonstrating its suitability for embedded and real-time diagnostic platforms.

By integrating commercial PCR tubes, a low-cost CMOS sensor, and a lightweight detection algorithm, the system enables stable fluorescence acquisition without relying on expensive hardware or computational resources. The ROI setting method also allows consistent alignment without repeated calibration, offering advantages in both structural simplicity and system scalability.

This approach can be applied across various use cases such as mobile PCR platforms, field-deployable diagnostic kits, and infectious disease response systems, contributing to the practical dissemination of cost-effective, high-efficiency molecular diagnostics. Furthermore, although the current implementation is based on a 5 × 5 layout, the proposed ROI detection method can be readily extended to larger formats, such as 8 × 12 or standard 96-well plates, with minor adjustments to the filter size and optical configuration.

## Figures and Tables

**Table 1:** Structural consistency and positional accuracy of the lid hole and optical window.

	Area Mean	Area Std/m	Center Std	Center Max Dev
Led holes	57,241.45	0.024	2.08	9.0 (0.172 mm)
Inner circles	17,264.93	0.039	3.24	15.5 (0.296 mm)

**Table 2:** Analysis Results of Tube Position Displacement.

Measurement Method	Maximum Position Deviation	Maximum Displacement	Standard Deviation	Mean Displacement	Variability Ratio (Std/Mean)
Manual measurement	15.5	15.182	3.363	5.927	0.567
Complex algorithm	22	19.235	3.88	7.682	0.505
Manual measurement–Complex algorithm		10.607	2.27	5.47	0.415

**Table 3:** Statistical results of the complex algorithm.

Analysis Item	Maximum Displacement (Pixel)
Maximum Displacement of Tube Position Across All 20 Images	24.18
Maximum Displacement Comparing Averaged Fluorescence Image Positions with Amplification Image Tube Positions	16.29
Maximum Displacement Comparing Generalized Tube Position Across All 20 Images	24.93

**Table 4:** Center and spacing statistics of the simple algorithm.

Methods	Result (Pixel)
1st center	3.6
Interval	Mean: 473 Deviation: 0.278
Real interval	477.93

## Data Availability

The raw data supporting the conclusions of this article will be made available by the authors on request.
